# Assessment of the impact of power business intelligence on adenoma detection rate: a prospective observational trial

**DOI:** 10.1186/s12876-025-03894-z

**Published:** 2025-04-19

**Authors:** Ya-Hui Hsu, Chia-Hsin Cheng, Ping-Hung Ko, Chia-Pei Tang, Chih-Wei Huang, Chih-Wei Tseng

**Affiliations:** 1 Division of Gastroenterology, Department of Internal Medicine, Dalin Tzu Chi Hospital, Buddhist Tzu Chi Medical Foundation, Chiayi, Taiwan; 2 School of Medicine, Tzu Chi University, Hualien City, Taiwan; 3 Department of Medical Research, Dalin Tzu Chi Hospital, Buddhist Tzu Chi Medical Foundation, Chiayi, Taiwan; 4 Dalin Tzu Chi Hospital Smart Medical Innovation Center, Buddhist Tzu Chi Medical Foundation, Chiayi, Taiwan

**Keywords:** Business intelligence, Colonoscopy, Adenoma detection rate, Feedback

## Abstract

**Background:**

Adenoma detection rate (ADR) is a key quality indicator in colonoscopy, with low ADRs linked to higher risks of post-colonoscopy colorectal cancers. Feedback to endoscopists has been shown to improve ADRs; however, many feedback systems lack automation and real-time interactivity. This study evaluates the effectiveness of Power Business Intelligence (Power BI) on ADR enhancement.

**Methods:**

This prospective observational study compared ADRs before (2021) and after (2022) the implementation of Power BI at Dalin Tzu Chi Hospital, Taiwan. Power BI automatically processed pathology data to calculate ADRs and provided real-time visual feedback on endoscopy quality indicators. A total of 4,306 colonoscopies performed by 10 endoscopists were analyzed. Logistic regression was employed to identify factors associated with ADR.

**Results:**

The overall ADR was high and comparable between the periods without and with Power BI (50.1% vs. 47.9%, *P* = 0.152). Individual performance was stable, though one low-performing endoscopist improved ADR by 20.0%. Adjusted multivariate analysis found no association between Power BI and ADR. Higher ADRs correlated with male gender (odds ratio [OR], 1.638; 95% confidence interval [CI], 1.438–1.864; *p* < 0.001), advanced age (OR, 1.642; CI, 1.439–1.875; *p* < 0.001), elevated BMI (OR, 1.642; CI, 1.439–1.875; *p* < 0.001), and positive stool occult blood (OR, 1.829; CI, 1.545–2.167; *p* < 0.001). Effective technical practices for improving ADRs included polyethylene glycol preparation (OR, 1.246; CI, 1.063–1.462; *p* = 0.007), water-method colonoscopy (OR, 1.321; CI, 1.134–1.538; *p* < 0.001), and withdrawal times ≥ 6 min (OR, 6.370; CI, 5.179–7.837; *p* < 0.001).

**Conclusions:**

The use of Power BI was not associated with a higher ADR at a high-performing institution but may benefit low-performing endoscopists. Efforts should target behavioral changes in modifiable technical factors to drive meaningful ADR improvements.

## Introduction

Colorectal cancer ranks as the third most commonly diagnosed malignancy worldwide, and screening program has contributed to its incidence and mortality [1, 2]. Among the screening methods, colonoscopy stands out as the most cost-effective, not only for its diagnostic capability but also for its potential in neoplasm removal, thereby reducing colon cancer-related deaths [3]. Despite its effectiveness, 2.9–9% of all colorectal cancers are post-colonoscopy colorectal cancers, occurring in patients who had undergone screening colonoscopy within the previous five years [4, 5, 6, 7]. The occurrence of post-colonoscopy colorectal cancers has been associated with a low adenoma detection rate (ADR), defined as the proportion of patients diagnosed with at least one adenoma [8]. Corley et al. reported that for each 1% increase in ADR, there was a corresponding 3% decrease in the risk of post-colonoscopy colorectal cancers and a 5% decrease in fatal post-colonoscopy colorectal cancers [5].

Several interventions have been shown to significantly improve ADR, including feedback for endoscopists, skill training, water-aided colonoscopy, second observations, advanced imaging techniques, and endoscopic devices [8, 9, 10, 11]. Feedback can be either active (hands-on training or personalized instruction) or passive (report cards or public reporting). A meta-analysis of 12 studies involving 78,355 participants demonstrated that both forms of feedback significantly improved ADR compared to no feedback [12]. Interestingly, even passive feedback led to a significant increase in ADR (pooled OR 1.43; *P* < 0.001). However, the substantial heterogeneity (*I*^2^ = 84%) indicates that variables such as study design, patient demographics, and feedback methods may influence outcomes differently across various contexts [12]. Furthermore, feedback mechanisms are not consistently automated, interactive, visually represented, or delivered in real time.

Power Business Intelligence (BI), an interactive data visualization software developed by Microsoft, is primarily designed for business intelligence applications. When integrated with data from the endoscopy room, it has the potential to provide real-time visual feedback to managers of endoscopic facilities. By analyzing longitudinal trends, this tool can help identify areas requiring improvement and establish critical benchmarks for assessing the quality of colonoscopies. Furthermore, it can deliver valuable feedback to endoscopists. To the best of our knowledge, no studies to date have examined the impact of Power BI on ADR.

Therefore, we conducted a prospective observational trial to evaluate the effect of passive feedback using Power BI on ADR. Additionally, we analyzed the endoscopist and patient factors associated with ADR to provide a comprehensive understanding of the variables influencing detection outcomes.

## Materials and methods

### Study design

**Fig. 1:**
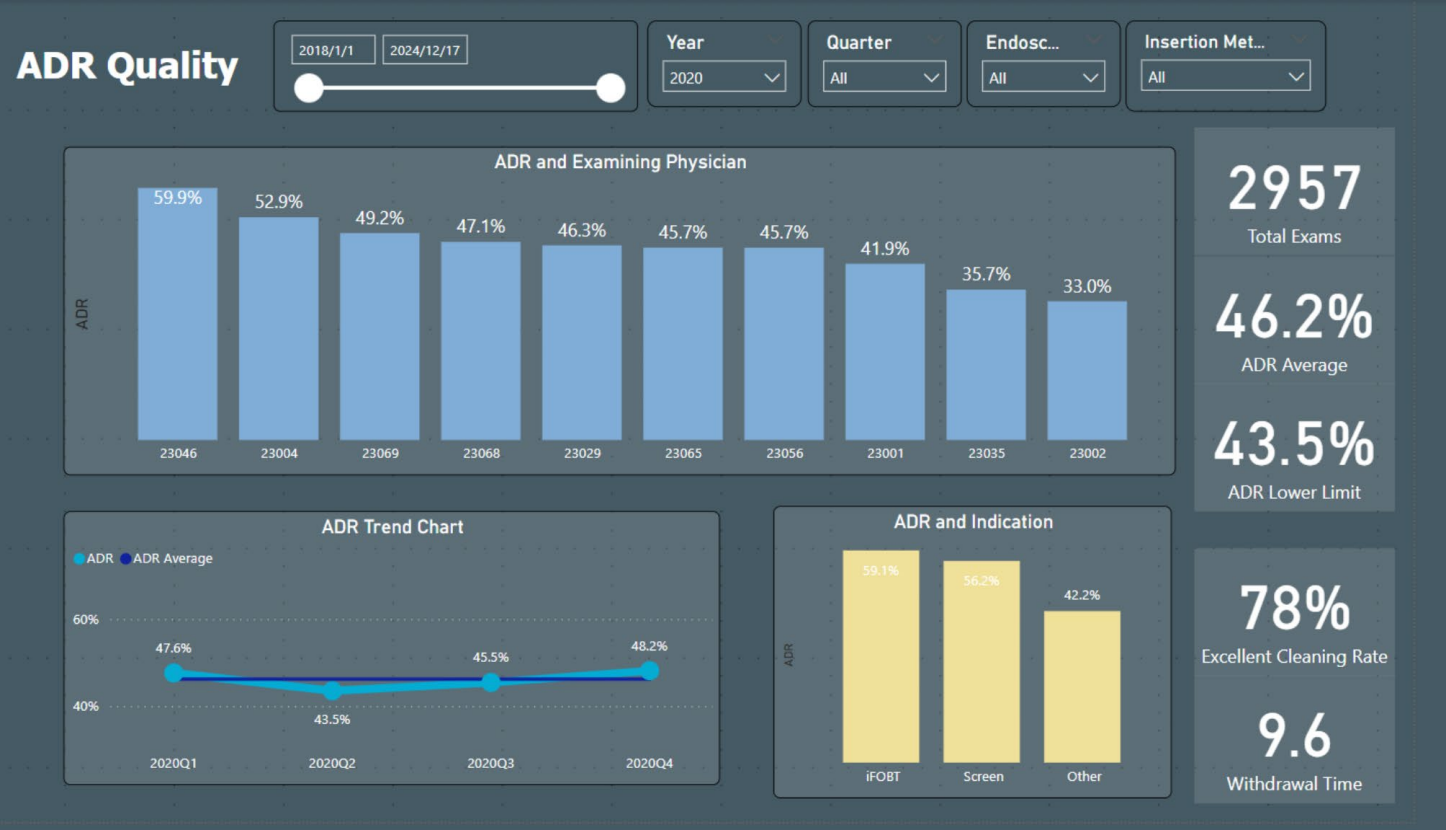
Power BI-based adenoma detection rate (ADR) Quality Dashboard: The dashboard visualizes ADR data segmented by time period, physician, and clinical indication, enabling endoscopists to monitor their performance and benchmark it against their peers

This study was conducted from January to December 2022 at Dalin Tzu Chi Hospital in Chiayi, Taiwan. In January 2022, we implemented a Business Intelligence system (Power BI, Microsoft) to monitor colonoscopy quality. Power BI was used to identify adenomas in pathology reports and calculate the adenoma detection rate. This system automatically generates a visual interface to assess metrics like polyp detection rate, adenoma detection rate, bowel preparation quality, and withdrawal time from colonoscopic reports. Real-time data were displayed on screens in the endoscopy room, allowing endoscopists to view and compare performance metrics with their peers (Fig. 1). Throughout 2022, Power BI was consistently employed to display colonoscopy quality data on screens within the endoscopy room. The Power BI visual report is available online upon individual login.

**Fig. 2:**
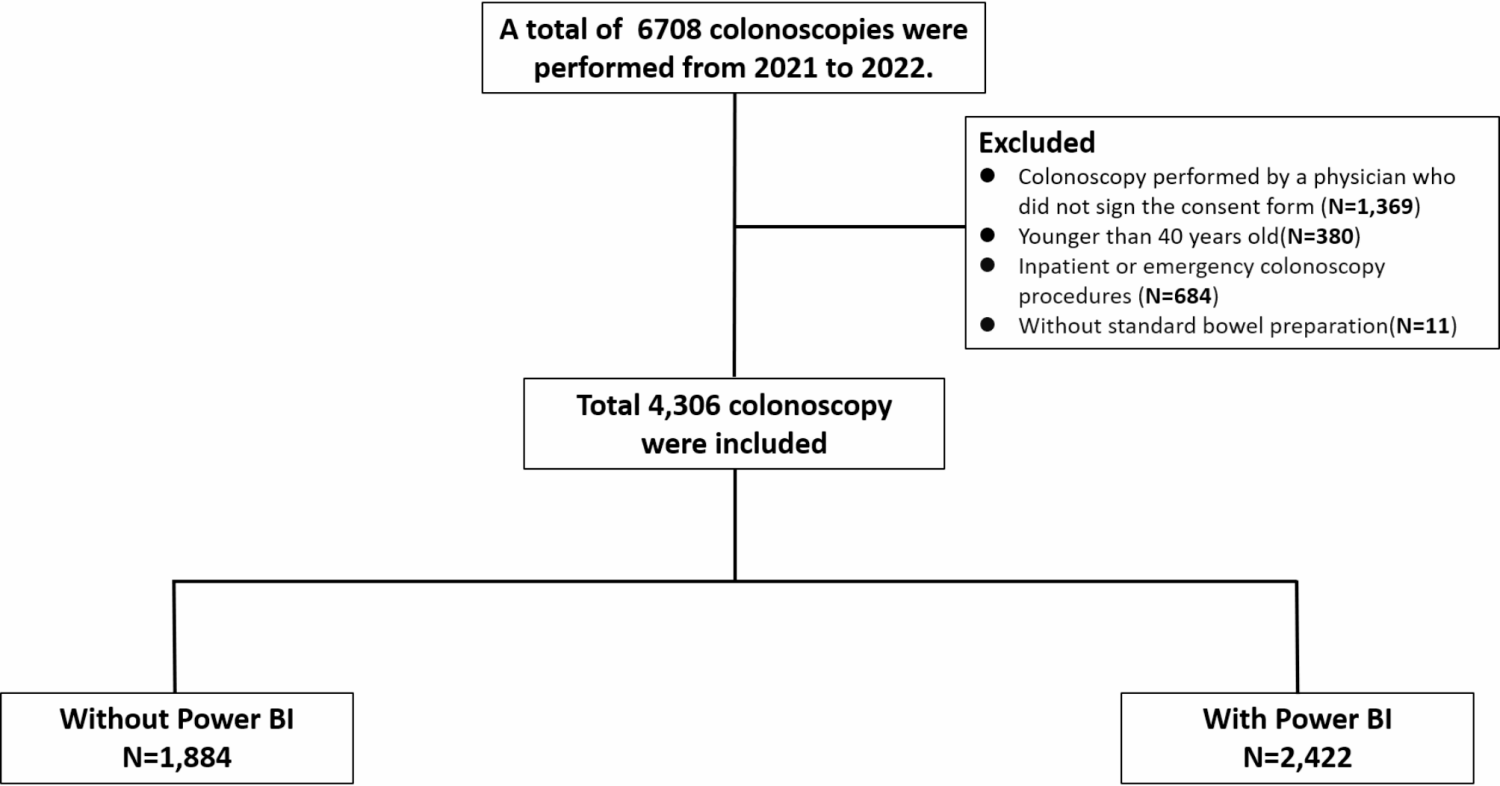
Flow diagram of inclusion criteria and group allocation

To evaluate the effectiveness of Power BI, data from 2021 served as the control group. The intervention data collected in 2022 were gathered prospectively following the implementation of Power BI, whereas the comparative control group data from 2021 were historical and collected retrospectively from electronic health records. A comparative analysis was performed, comparing the data from 2022 (With Power BI group) with the data from 2021 (Without Power BI group). This study encompassed outpatient elective colonoscopy procedures utilizing standard bowel preparation. Exclusions included patients younger than 40 years old, inpatient or emergency colonoscopy procedures, procedures conducted without standard bowel preparation, and those performed by physicians who had not signed the consent form (Fig. 2).

This study adhered to the ethical principles of the 1975 Declaration of Helsinki. Approval was obtained from the Ethics Committee of Dalin Tzu Chi Hospital (approval number B11101008). Ten out of 13 board-certified colonoscopists at our hospital agreed to participate in performance monitoring, with informed consent obtained. Five senior endoscopists have extensive expertise, having performed 5,000 to 6,000 colonoscopy procedures over 20 to 25 years of practice. In contrast, the other five novice endoscopists have completed around 1,000 to 2,000 procedures, with 10 to 15 years of experience.

### Power BI feedback intervention

Implemented throughout 2022, the Power BI feedback intervention provided real-time performance updates as pathology results became available. This system ensured continuous access to critical colonoscopy metrics, including ADR, polyp detection rates, bowel preparation quality, and withdrawal times. Endoscopists received both institutional and individual performance data (Fig. 1). Institutional data provided an overview of colonoscopy quality indices across the hospital, while individual performance data enabled endoscopists to assess their own ADR in comparison to their colleagues. Two types of feedback were provided to the endoscopists. Real-time visualizations of these metrics were displayed on monitors within endoscopy rooms, facilitating immediate quality assessment during procedures. Additionally, the system remained accessible via a secure online dashboard through individualized logins. This dual feedback approach aimed to enhance awareness and promote performance optimization. No concurrent interventions, such as specialized training or equipment upgrades, were introduced, ensuring an independent evaluation of the system’s impact.

### Outcome variables

The primary objective of this study was to evaluate the impact of Power-BI on the ADR. Demographic factors extracted from the electronic records included age, sex, height, weight, indications for colonoscopy, and histories of abdominal or pelvic surgery, alcohol consumption, and smoking. Procedural data were also collected, covering the use of sedation, cecal insertion time and method, withdrawal time, total procedure time, and the quality of bowel preparation. The latter was assessed using the Boston Bowel Preparation Scale (BBPS), a validated scoring system that evaluates bowel cleanliness by summing segmental scores (ranging from 0 to 3) for three different colonic segments: the right, transverse, and left colon [13]. Adequate bowel preparation was defined as a total BBPS score of ≥ 6 [14]. Subsequent analyses were conducted based on this threshold.

Body mass index (BMI) was calculated using the formula: BMI = body weight (kg) / [height (m)]^2^. According to Taiwan’s recommendation, a BMI of 24 or higher is classified as overweight [15]. The elderly population was defined as individuals aged 65 years and older [16]. The endoscopist recorded procedure times using a designated button at three points: start of the exam, colonoscope reaching the cecum, and completion. The total procedure time (in minutes) was calculated from the start to the end of the examination. Insertion time was measured from the beginning of the procedure to the point when the colonoscope reached the cecum. Withdrawal time was recorded from the start of scope withdrawal at the cecum to the completion of the procedure. An adequate withdrawal time for colonoscopy was defined as a minimum of 6 min [17].

### Preparation and procedure for colonoscopy

All colonoscopy preparations followed a standard bowel preparation regimen. Standard bowel preparation was defined as a split-dose regimen using 2 L polyethylene glycol (PEG) or two sachets of sodium picosulfate/magnesium citrate (SPMC) plus bisacodyl. Patients took 10 mg of bisacodyl at noon, then consumed 1 L of polyethylene glycol (PEG) or one sachet of sodium picosulfate/magnesium citrate (SPMC) between 7 pm and 9 pm. The remaining dose was taken at 6 am on the procedure day. Colonoscopies used standard Olympus colonoscopes (CF-H260 and CF-HQ290). Patients started in the left lateral decubitus position. Successful cecal intubation was confirmed by visualizing the appendiceal orifice or ileocecal valve.

### Statistical analysis

All statistical analyses were performed using IBM SPSS Statistics software, version 23.0 (IBM Corp., Armonk, NY, USA). Baseline characteristics and colonoscopic outcomes were summarized as frequencies and percentages for categorical variables, while continuous variables were presented as medians with interquartile ranges (IQR) due to their non-normal distribution. Group comparisons for categorical variables were conducted using the Chi-square test or Fisher’s exact test, as appropriate. The Mann-Whitney U test was used to compare continuous variables between groups. To determine independent predictors of ADR, univariate and multivariate logistic regression analyses were conducted utilizing a stepwise elimination procedure. Statistical significance was defined as *P* < 0.05. Multiple comparisons were adjusted using the Holm-Bonferroni method. Model goodness-of-fit was evaluated using the Hosmer-Lemeshow test.

## Results

### Baseline characteristics

A total of 4,306 colonoscopy exams were performed by 10 physicians (Fig. 2). There were 1,884 procedures in the “Without Power BI” group and 2,422 in the “With Power BI” group (Table 1). The median age of participants was higher in the “Without Power BI” group (62 years, IQR 53–70) compared to the “With Power BI” group (61 years, IQR 52–69; *P* = 0.001). A greater proportion of patients in the “Without Power BI” group underwent bowel preparation with PEG (77.6% v*s*. 71.9%; *P* < 0.001). Additionally, stool occult blood positivity was more common in the “Without Power BI” group (22.6% v*s*. 16.4%; *P* < 0.001). Both groups were similar in terms of gender distribution, BMI, smoking, drinking, or full sedation during colonoscopy.

**Table 1:** Baseline characteristics and outcomes

Baseline characteristics	Total (*n* = 4,306)	Without Power BI (*n* = 1,884)	With Power BI (*n* = 2,422)	*P* value
Male, *n* (%)	2,177 (50.6)	963 (51.1)	1,214 (50.1)	0.519^b^
Age (years), median (IQR)	61 (52–69)	62 (53–70)	61 (52–69)	0.001^a^
BMI (kg/m^2^), median (IQR)	24.9 (22.4–27.5)	24.8 (22.4–27.6)	24.9 (22.4–27.4)	0.794^a^
Fully sedated colonoscope, *n* (%)	2,723 (63.2)	1,177 (62.5)	1,546 (63.8)	0.359^b^
Bowel preparation with PEG, *n* (%)	3,204 (74.4)	1462 (77.6)	1742 (71.9)	< 0.001^b^
Smoking, *n* (%)	609 (14.1)	268 (14.2)	341 (14.1)	0.892^b^
Drinking, *n* (%)	512 (11.9)	227 (12.0)	285 (11.8)	0.777^b^
Indication for stool OB positive, *n* (%)	822 (19.1)	425 (22.6)	397 (16.4)	< 0.001^b^
**Outcomes of colonoscopy**				
Adenoma detection rate, %(*n*)	48.8 (2,102)	50.1 (943)	47.9 (1,159)	0.152^b^
Adenomas number per colonoscopy, mean (IQR)	0.97 (0–1)	0.90 (0–1)	0.84 (0–1)	0.112^a^
Polyp detection rate, %(*n*)	55.9 (2,406)	58.6 (1104)	53.8 (1302)	0.002^b^
Sessile serrated adenoma detection rate %(*n*)	0.9 (40)	0.8 (16)	1.0 (24)	0.631^b^
Cecal intubation rate, *n* (%)	4,256 (98.8)	1,858 (98.6)	2,398 (98.9)	0.520 ^b^
Cecal insertion time (minutes), median (IQR)	5 (3–8)	5 (3–8)	5 (3–8)	0.686^a^
Withdrawal time (minutes), median (IQR)	8 (6–12)	8 (6–12)	8 (6–11)	0.005^a^
Total procedure time (minutes), median (IQR)	14 (10–19)	15 (10–20)	14 (10–19)	0.122^a^
BBPS score ≥ 6, *n* (%)	3,633 (85.9)	1,617 (85.8)	2,083 (86.0)	0.895 ^b^

(a) Mann-Whitney *U* test, (b) Chi-squared test,

*Other include Diagnostic, Surveillance, Screening

SD, standard deviation; BMI, body mass index; BBPS, Boston Bowel Preparation Scale; BI, business intelligence; IQR, interquartile range; PEG, polyethylene glycol; OB+, stool occult blood

### Colonoscopy outcomes

**Fig. 3:**
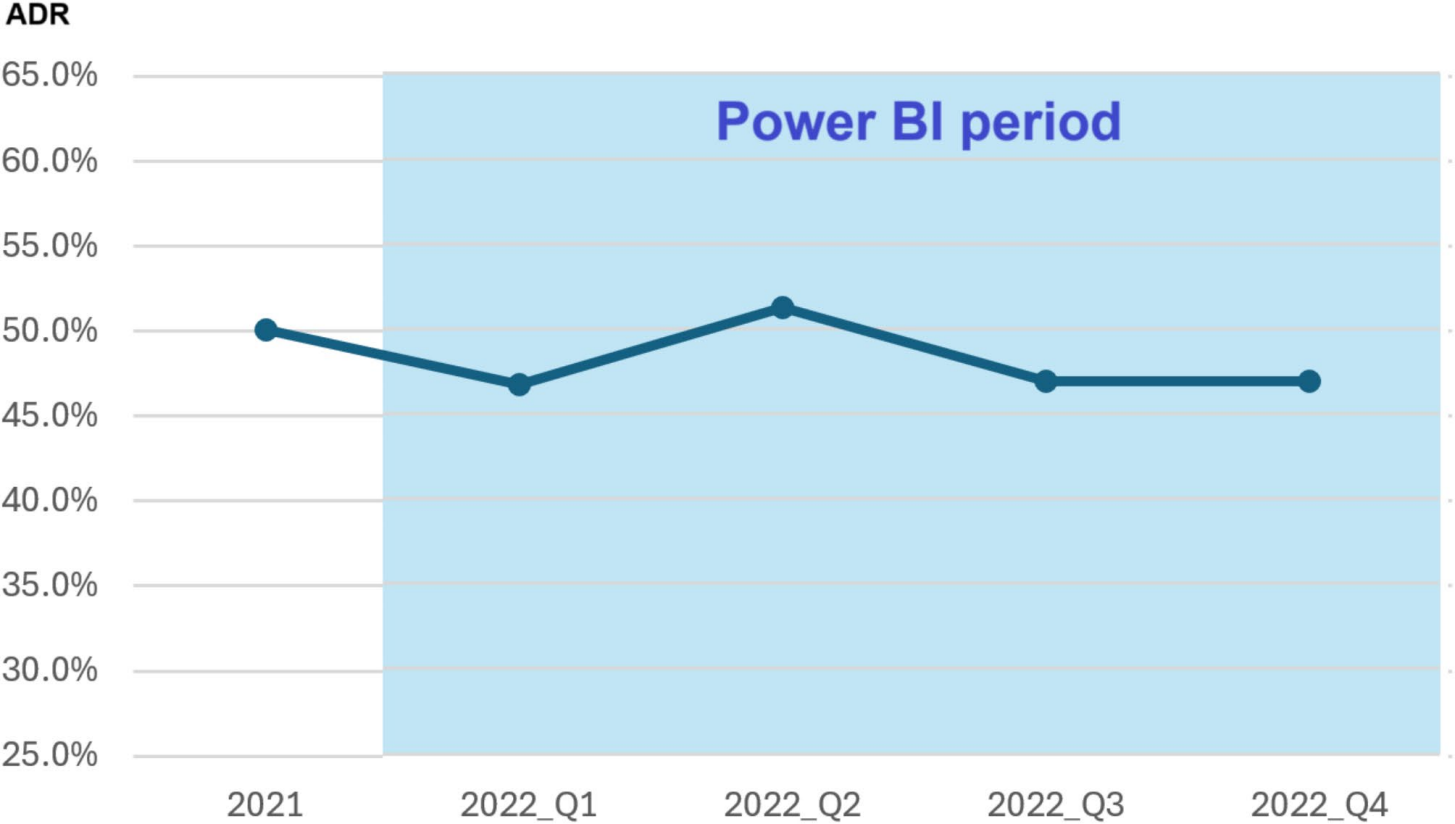
Adenoma detection rate over time with power BI implementation

The primary endpoint, ADR, was comparable between the two groups, with a detection rate of 50.1% observed in the “Without Power BI” group versus 47.9% in the “With Power BI” group (*P* = 0.152). Figure 3 illustrates the quarterly ADR trends before and after the implementation of Power BI. During the baseline period in 2021, the ADR was approximately 50.1%. Following the introduction of Power BI, the quarterly ADR remained stable, ranging from 46.7 to 51.3%, without significant variation.

**Fig. 4:**
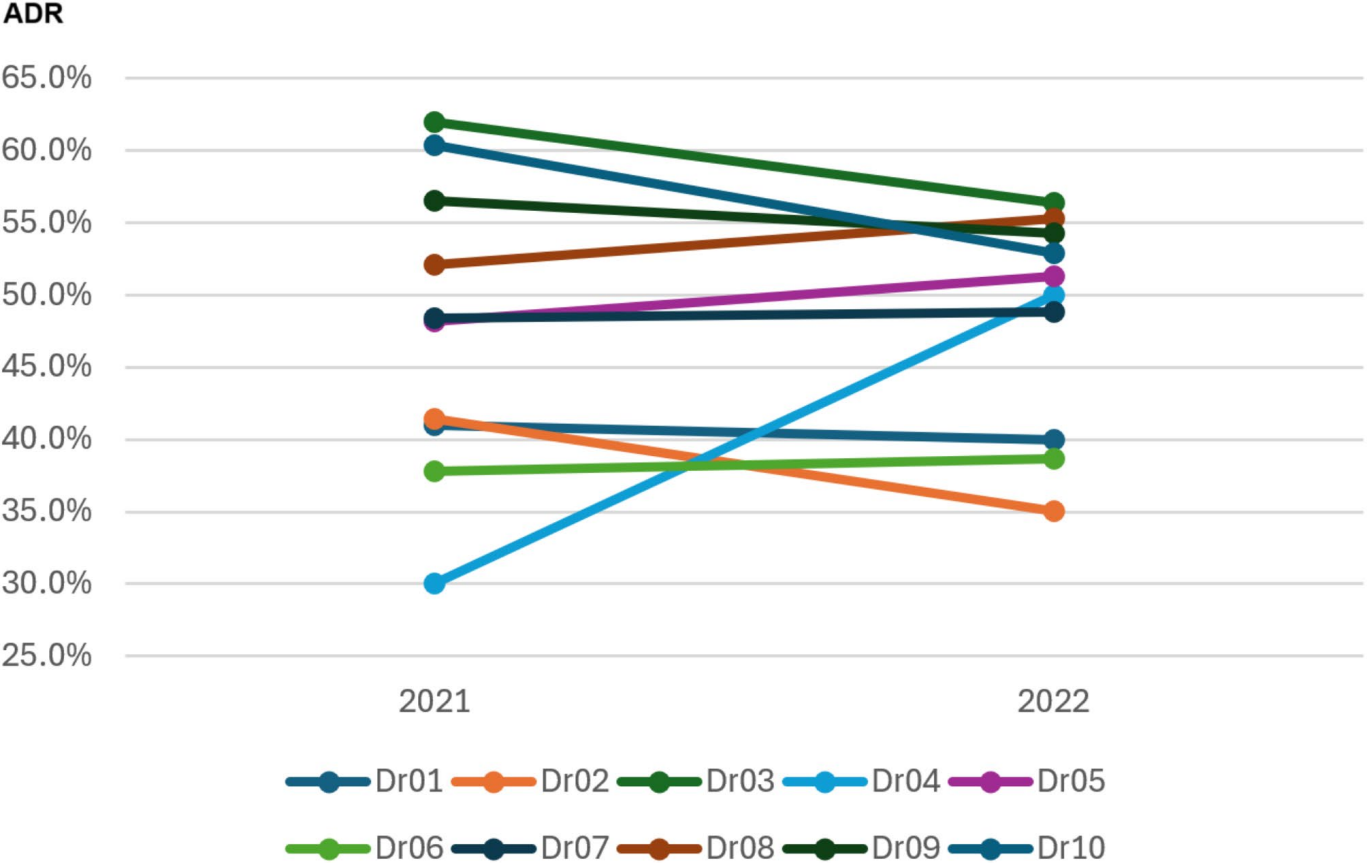
Adenoma detection rate trends by physician (2021–2022)

Figure 4 illustrates the ADR trends for 10 individual endoscopists across 2021 and 2022. Despite the implementation of Power BI in 2022, the overall ADR changes varied among endoscopists, without statistically significant change observed at the group level. Among the 10 endoscopists, five (Dr01, Dr02, Dr03, Dr09 and Dr10) demonstrated a decline in ADR from 2021 to 2022, with reductions ranging from − 1.0% (Dr01) to -7.5% (Dr10). Conversely, three endoscopists (Dr04, Dr05, and Dr08) experienced increases in ADR, with the most substantial improvement observed in Dr04, who showed a 20.0% increase (30.0% in 2021 to 50.0% in 2022). The remaining endoscopists (Dr06, and Dr07) exhibited stable ADR performance, with minimal changes (< 1%). Subgroup analysis confirmed that ADR remained consistent across experience levels, despite Power BI implementation. The ADR for senior physicians was 49.5% without Power BI and 46.7% with it (*P* = 0.147), while for novice physicians, it was 51.1% and 49.9%, respectively (*P* = 0.678).

Similarly, the mean number of adenomas per colonoscopy did not differ significantly between groups (mean 0.90, IQR 0–1 in the “Without Power BI” group compared to 0.84, IQR 0–1 in the “With Power BI” group; *P* = 0.112). Secondary outcomes revealed that polyp detection rates were notably higher in the “Without Power BI” group (58.6% versus 53.8%; *P* = 0.002). Sessile serrated adenoma detection rates were uniformly low and demonstrated no significant difference (0.8% versus 1.0%; *P* = 0.631).

The procedure outcomes consistently demonstrated high cecal intubation rates of approximately 98% across all groups (*p* = 0.520). The median cecal insertion time was 5 min in all groups (*p* = 0.686). Notably, withdrawal times were longer in the “Without Power BI” group (median: 8 min, IQR: 6–12) compared to the other group (median: 8 min, IQR: 6–11; *p* = 0.005). The median total procedure times ranged from 14 to 15 min without significant variation (*p* = 0.122). The proportion of cases achieving a BBPS score ≥ 6 was similar between groups, with rates of 85.8% versus 86.0% (*p* = 0.895).

### Factors associated with ADR

Table 1 presents significant differences in factors such as age, indication for stool occult blood positivity, withdrawal time, and bowel preparation drug that may be associated with ADR. To assess the impact of PowerBI on ADR, multivariable logistic regression analysis was applied to adjust for these factors and determine which factors are associated with ADR (Table 2).

**Table 2:** Univariate and multivariate logistic regression analyses of factors associated with adenoma detection rate (ADR) (*N* = 4,306)

Variable	ADR (%)	Univariate logistic regression		Multivariate logistic regression#	
		OR (95% CI)	*P* value	OR (95% CI)	*P* value
**Power BI usage**					
Before (2021)	50.1	Ref		Ref	
After (2022)	47.9	0.916 (0.812, 1.033)	0.152	0.994 (0.873–1.133)	0.931
**Gender**					
Female	42.0	Ref		Ref	
Male	55.4	1.716 (1.521, 1.936)	< 0.001	1.638 (1.438–1.864)	< 0.001
**Age**					
< 65	43.9	Ref		Ref	
≥ 65	56.1	1.632 (1.443, 1.845)	< 0.001	1.642 (1.439–1.875)	< 0.001
**BMI (kg/m** ^**2**^ **)**					
<24	43.2	Ref		Ref	
≥24	50.6	1.348 (1.170, 1.553)	< 0.001	1.269 (1.089–1.478)	0.002
**Indication**					
Others*	46.2	Ref		Ref	
Positive stool occult blood	59.7	1.725 (1.478, 2.013)	< 0.001	1.829 (1.545–2.167)	< 0.001
**Method for the colonoscope**					
Air	45.4	Ref		Ref	
Water	59.7	1.377 (1.246, 1.436)	< 0.001	1.321 (1.134–1.538)	< 0.001
**Preparation drug**					
SPMC	43.5	Ref		Ref	
PEG	50.7	1.355 (1.163, 1.532)	< 0.001	1.246 (1.063–1.462)	0.007
**Withdrawal time (min)**					
<6	17.0	Ref		Ref	
≥6	56.5	6.344 (5.232, 7.691)	< 0.001	6.370 (5.179–7.837)	< 0.001
**BBPS score**					
0–5	48.2	Ref			
6–9	51.0	1.159 (0.976, 1.376)	0.093	**-**	
**Fully sedated colonoscopy**					
Non- sedated	49.9	Ref			
Fully sedated	48.2	0.933(0.825,1.057)	0.275	-	
**Experience**					
Novice	50.4	Ref			
Senior	47.9	0.905(0.799,1.026)	0.119		

BBPS, Boston Bowel Preparation Scale; BMI, body mass index; CI, confidence interval; OR, odds ratio; SPMC, sodium picosulfate/magnesium citrate; *Other include Diagnostic, Surveillance, Screening

# Multivariable analysis was conducted by adjusting for Power BI usage, gender, age, and factors with a *P*-value < 0.05 in univariate analysis

To control Type I error inflation, the Holm-Bonferroni method was applied, adjusting the significance threshold as α’ = 0.05 / (8 - rank + 1) for each ordered *p*-value. After correction, all variables remained statistically significant. The Hosmer-Lemeshow test (χ^2^ = 2.574, df = 8, *P* = 0.958) confirmed the model’s adequacy, indicating a good fit to the observed data. After analysis, PowerBI is not an important factor affecting ADR. However, the gender (odds ratio[OR], 1.638;95% confidence interval [CI], 1.438–1.864; *p* < 0.001), age ≥ 65 years (OR, 1.642; 95% CI, 1.439–1.875; *p* < 0.001), BMI ≥ 24 kg/m^2^ (OR, 1.269; 95% CI, 1.089–1.478; *p* = 0.002), indication positive stool occult blood (OR, 1.829; 95% CI, 1.545–2.167; *p* < 0.001), method of colonoscopy with water use (OR, 1.321; 95% CI, 1.134–1.538, *p* < 0.001), bowel preparation with PEG (OR, 1.246; 95% CI, 1.063–1.462, *p* = 0.007), and a withdrawal time ≥ 6 min (OR, 6.370; 95% CI, 5.179, 7.837; *p* < 0.001) are important indicators that affect the adenoma detection rate during colonoscopy.

## Discussion

In this study, the implementation of Power BI on reporting desktops did not result in an improvement in ADRs. Quarterly ADR trends remained consistent, ranging from 46.7 to 51.3%. No significant changes observed in individual endoscopist performance, except for one physician who demonstrated a notable 20.0% improvement (from 30.0% in 2021 to 50.0% in 2022). After adjusting for confounding factors, multivariate analysis revealed no association between Power BI usage and adenoma detections. Adenoma detections were associated with patient factors such as male gender, advanced age, elevated BMI, and positive stool occult blood, as well as technical factors like PEG preparation, water-method colonoscopy, and withdrawal times ≥ 6 min.

Advancements in artificial intelligence (AI) and BI tools present promising opportunities for refining feedback mechanisms in endoscopy. However, the effectiveness of these technologies in improving ADRs and overall endoscopic performance varies across studies [18, 19, 20]. Our study found no significant ADR improvement with Power BI usage (50.1% vs. 47.9%), suggesting that passive visualization tools may insufficiently address detection challenges. Similarly, a BI tool integrated into the Netherlands’ Trans.IT database showed limited impact on ADR [18]. Their system supports real-time dashboards and custom reports for anonymized data, maintaining high-quality CRC-screening colonoscopy rates (ADR 63.6% in 2016; 53.8% in 2020), yet without notable improvements [18]. The NED-APRIQOT trial, analyzing over 70,000 procedures with automated monthly performance reports from United Kingdom National Endoscopy Database, also found no significant gains in adjusted polyp detection performance (7%, 95% CI, -1–14%; *P* = 0.08) [19, 21]. These outcomes illustrate the challenges of feedback from generalized reporting systems in driving performance improvements.

In contrast to the above studies, Lim et al. reported on the effectiveness of automated natural language processing software (EndoMineR) in improving ADRs [20]. This software identified colonic adenomas from pathology reports and and provided personalized feedback to endoscopists every six months. Over an eight-year period encompassing 39,359 colonoscopies, the study observed a significant increase in ADR, rising from a baseline of 12.7–21.0%. Such increase was partly attributed to greater use of the Endocuff device [20]. Additionally, their findings underscore the role of automated reporting in detecting performance gaps. Compared to our cohort and the Netherlands’ Trans.IT database, Lim et al. reported the lowest baseline ADR, suggesting the potential value of generalized reporting systems for institutions with lower baseline ADRs. The limited impact of Power BI may be due to information fatigue and the lack of personalized intervention. These findings suggest that feedback frequency, real-time visualization, and baseline ADR play crucial roles in determining the effectiveness of reporting systems in improving ADR. For endoscopists with lower performance, enhancing Power BI’s internal data analysis to identify performance gaps, combined with personalized training, may significantly improve their skills.

The lack of significant improvement in ADR after Power BI feedback in our study may reflect the high baseline performance of our endoscopists. The baseline ADR within our hospital was approximately 50%, with a single outlier at 30% (Fig. 4). Prior studies have reported that ADR improvements were significantly greater among endoscopists with the lowest baseline ADRs compared to those with higher initial rates [22, 23, 24]. A meta-analysis of 12 studies (1 RCT and 11 non-RCTs) involving 154 endoscopists and 33,184 colonoscopies reported a pooled pre-feedback ADR of 30.5%, increasing to 36.0% post-feedback (risk ratio[RR], 1.21; 95% CI, 1.09–1.34) [10]. However, the included studies exhibited substantial heterogeneity (I^2^ =87%) [10]. Subgroup analyses indicated that endoscopists with low baseline ADRs (26–30%) experienced the most substantial improvement (RR, 1.62; 95% CI, 1.18–2.23), followed by moderate performers (ADR 30–35%, RR, 1.19; 95% CI, 1.11–1.29). High baseline ADRs (> 35%), however, did not exhibit statistically significant benefit (RR, 1.06; 95% CI, 0.99–1.13) [10]. In our study, the sole colonoscopist who demonstrated a notable 20% increase ADR (from 30.0 to 50.0%) was a lower performer. These findings suggest that while institutions with high baseline ADRs may experience limited overall gains, targeted feedback strategies for low-performing endoscopists may yield meaningful improvements.

We did not observe a direct improvement in other colonoscopy quality indicators. This could mean that either the quality of endoscopy was already high, or the Power BI can’t change the behavior. Information overload may partly explain why Power BI has limited impact on ADR improvement. Power BI enables endoscopists to monitor their performance in real time and receive frequent updates within endoscopy rooms. However, as feedback was delivered at least weekly, the high frequency may have contributed to information overload. A study with 528 healthcare providers found that every additional public health message per week reduced recall odds by 41.2% [25]. Frequent and repetitive information across multiple channels can disrupt effective communication [26, 27]. This issue is particularly critical in ADR improvement, where behavioral changes require weeks or months. Excessive feedback can create frustration due to the perceived lack of progress, thereby diminishing the motivation for behavioral change. Further research is needed to explore optimal feedback strategies and effective communication approaches.

Improving ADR is particularly beneficial for physicians with lower baseline rates, as it reduces post-colonoscopy colorectal cancer risk. A recent observational study found that for physicians with a baseline ADR below 26%, increases over time significantly correlated with lower colorectal cancer risk, unlike those with stable ADR [28]. However, for physicians with a baseline ADR of 26% or higher, improvements were not linked to colorectal cancer risk reduction [28]. Our study demonstrates that Power BI effectively enhances ADR for lower-performing endoscopists. Building on these findings, future research should focus on evaluating the impact of Power BI in helping underperforming physicians attain acceptable ADR levels and assess its potential in reducing post-colonoscopy colorectal cancer risk.

In this study, multivariate analysis, adjusted for confounding factors, further demonstrated no significant association between the use of Power BI and ADRs. Additionally, we identified factors associated with adenoma detection. Consistent with previous studies, higher ADRs were linked to patient characteristics, including male gender, advanced age, elevated BMI, and positive fecal occult blood tests [29, 30, 31, 32]. These findings underscore the importance of targeted screening strategies and lifestyle modification [32, ].

In contrast to the above patient factors, the modifiable technique factors were more important because they could be provided by health care providers [9]. In our study, a withdrawal time of ≥ 6 min had the greatest impact on ADR, with an OR of 6.370 (95% CI, 5.179–7.837; *p* < 0.001). Sufficient withdrawal time enables colonoscopists to conduct a precise and detailed colon inspection. Research indicates that longer withdrawal times facilitate thorough colon inspections, enhancing ADRs. Each additional minute of withdrawal time increased adenoma detection odds by 6% (OR, 1.06; 95% CI, 1.02–1.10; *p* = 0.004) [33], though this effect diminishes beyond 10–13 min [33, 34]. Given the high OR observed in our study, behavioral modifications to extend withdrawal time may produce the most significant improvements in ADR.

Water colonoscopy techniques, particularly water exchange, have been shown to significantly improve adenoma detection rates by enhancing bowel cleanliness [9, 35]. Our findings further support the efficacy of water colonoscopy techniques (OR: 1.321; 95% CI: 1.134–1.538; *p* < 0.001). However, due to limitations in clinical documentation, it remains uncertain whether colonoscopists strictly adhered to water exchange or water immersion. Further research is warranted to elucidate the distinct effects of these methods on ADR within the context of Power BI feedback. Additionally, our results favor bowel preparation with PEG, yielding an OR of 1.246 (95% CI: 1.063–1.462; *p* = 0.007). While previous meta-analyses demonstrated that SPMC provides bowel-cleansing efficacy comparable to PEG, the effect direction leaned toward PEG (RR: 0.93; 95% CI: 0.86–1.01; *p* = 0.07) [36]. Based on these findings, in addition to extending withdrawal time, adopting water colonoscopy techniques and utilizing PEG for bowel preparation should be considered as viable modifications to clinical practice.

AI has demonstrated significant potential in real-time adenoma detection, contributing to the enhancement of the adenoma detection rate (ADR) [37, 38, 39]. Future research could investigate the integration of AI with Power BI to facilitate comprehensive endoscopic procedure analysis. Such an integration would enable AI-driven evaluations of withdrawal time and field-of-view coverage, including blind spot identification, while providing real-time feedback on the necessity of re-examining specific areas. Additionally, AI-generated personalized reports could highlight anatomical regions requiring more thorough inspection. This synergy may transform Power BI from a conventional data monitoring tool into a sophisticated platform for quality assurance and performance optimization in endoscopy.

The limitations of this study must be acknowledged. First, the non-randomized study design may introduce potential confounding factors. Although multivariable analysis was applied to mitigate these effects, the inherent limitations of observational studies remain. Thus, future multi-institutional randomized controlled trials are essential to substantiate the effectiveness of Power BI in improving adenoma detection rates. Second, the data are institution-specific, which may limit the generalizability of the findings to other settings with differing baseline ADRs or medical systems. Expanding the application of this system to additional hospitals and addressing its broader impact is necessary. Third, the study does not investigate why Power BI failed to influence behavior, particularly in relation to its potential limitations, such as the adequacy of its feedback mechanism or the variability in endoscopists’ acceptance of feedback. Furthermore, it does not explore whether enhancements to its interface or functionality—particularly through a more personalized design—could improve its effectiveness. Addressing these gaps warrants further research. Fourth, the comparison of 2022 intervention data with 2021 historical controls introduces the potential for selection and information bias. This study utilized multivariate adjustment to control for confounders related to patient demographics, referral patterns, and procedural practices. However, residual confounding factors, such as family history colorectal cancer and education level, may still be present, warranting further research and cautious interpretation of the findings. Fifth, the one-year follow-up in this study may not fully capture long-term ADR trends. Since ADR evolves over time, a long-term study would help assess the sustained effects of Power BI on endoscopist behavior.

## Conclusions

The study demonstrates that the use of Power BI was not associated with a higher ADR at a high-performing institution. However, the observed improvement in ADR for a low-performing endoscopist highlights the potential of feedback tools to benefit individual practitioners with lower baseline ADRs. Multivariate analysis revealed no significant association between Power BI usage and ADRs. However, it identified key determinants of ADR, including patient-related factors (e.g., male gender, advanced age, elevated BMI, and positive fecal occult blood tests) and procedural factors (e.g., adequate withdrawal time, water-assisted techniques, and PEG bowel preparation). These findings provide actionable targets for clinical practice improvement. Nonetheless, this single-center, non-randomized study has limitations, including incomplete control of confounding factors. Future multicenter randomized controlled trials are needed to validate the effect of Power BI on adenoma detection rates.

## Data Availability

The datasets generated and/or analyzed during the present study are not publicly available due to privacy restrictions imposed by the hospital database. However, they may be obtained from the corresponding author upon reasonable request.

## Declarations

ADR: Adenoma detection rate
BBPS: Boston Bowel Preparation Scale
BMI: Body mass index
BI: Business intelligence
CI: Confidence interval
IQR: Interquartile range
OR: Odds ratio
PEG: Polyethylene Glycol
SPMC: Sodium Picosulfate/Magnesium Citrate
WE: Water exchange
